# Probiotics: The Health Boosters

**DOI:** 10.4103/0974-2077.58530

**Published:** 2009

**Authors:** Rajiv Saini, Santosh Saini, Sugandha

**Affiliations:** *Department of Periodontology and Oral Implantology, Rural Dental College, Loni, Maharashtra, India. E-mail: drperiodontist@yahoo.co.in*; 1 *Department of Microbiology, Rural Dental College, Loni, Maharashtra, India*; 2 *Department of Prosthodontics, Rural Dental College, Loni, Maharashtra, India*

Sir,

The World Health Organization and the Food and Agriculture Organization of the United Nations have defined probiotics as "live microorganisms, which, when administered in adequate amounts, confer a health benefit on the host." They are also called "friendly bacteria" or "good bacteria." The concept of probiotics arose at the turn of the 20^th^ century from a hypothesis first proposed by Noble Prize-winning Russian scientist, Elie Metchnikoff. Most probiotics fall into the group of organisms known as lactic acid-producing bacteria and are normally consumed in the form of yogurt, fermented milks, or other fermented foods.[1] Prebiotics are nondigestible food ingredients that selectively stimulate the growth and/or activity of beneficial microorganisms already in the human colon, and when mixed with probiotics, form synbiotics.[2]

For an organism to be considered as probiotic, the following criteria need to be fulfilled:[3] (1) It should be isolated from the same species as its intended host, (2) It should have a demonstrable beneficial effect on the host, (3) It should be nonpathogenic, (4) It should be able to survive transit through the gastrointestinal tract, and (5) On storage, large number of viable bacteria must be able to survive prolonged periods. Most probiotics are bacteria similar to those naturally found in the human gut, especially in those of breastfed infants (who have natural protection against many diseases). Frequently, the bacteria come from two groups, *Lactobacillus* or *Bifidobacterium*. Within each group, there are different species (for example, *Lactobacillus acidophilus* and *Bifidobacterium bifidus*), and within each species, different strains (or varieties). Some common probiotics such as *Saccharomyces boulardii* are yeasts, which are different from bacteria.

The physiological effects attributed to probiotic bacteria include: i) the reduction of gut pH, ii) production of some digestive enzymes and vitamins, iii) production of antibacterial substances, *e.g*., organic acids, bacteriocins, hydrogen peroxide, diacetyl acetaldehyde, lactoperoxidase system, lactones, and other unidentified substances, iv) reconstruction of normal intestinal microflora after disorders caused by diarrhoea, antibiotic therapy, and radiotherapy, v) reduction of cholesterol level in the blood, vi) stimulation of immune functions, vii) suppression of bacterial infections, viii) removal of carcinogens, ix) improvement of calcium absorption, as well as ×) the reduction of fecal enzyme activity. [2] Mechanisms for the benefits of probiotics are not completely understood. However, some proposed mechanisms include;[3,4] (1) Adherence and colonization of the gut, (2) Suppression of growth or epithelial binding/invasion by pathogenic bacteria and production of antimicrobial substances, (3) Improvement of intestinal barrier function, (4) Controlled transfer of dietary antigens, and (5) Stimulation of mucosal and systemic host immunity.

Probiotics are generally considered safe.[3] As evidenced by epidemiological studies, bacteremia or sepsis from lactobacilli is extremely rare. Numerous probiotics have a long history of safe use and no health concerns have been recognized.[5]

Phyisicians need to be aware of the role of probiotics in health and during administration of antibiotics which alter the probiotic population. Measures to enhance their role, such as combination of probiotic bacteria with nutrient-dense foods like dairy products, should be recognized and studied.[6] Probiotic bacteria may offer an alternative to antibiotic therapy as a low-cost, low-risk method of protection from infection.[6] Probiotics can be included in foodstuffs through new technologies like microencapsulation and immobilized cell technologies. Further research in this area may offer exciting avenues in health care strategies.
